# T-staging of rectal cancer: Utility of single-shot turbo spin-echo diffusion-weighted imaging with T2-weighted images and fusion images

**DOI:** 10.1371/journal.pone.0249433

**Published:** 2021-04-21

**Authors:** Masaki Ogawa, Misugi Urano, Taku Takaishi, Hirohito Kan, Nobuyuki Arai, Hiroki Takahashi, Masayasu Hara, Miki Saito, Yuta Shibamoto

**Affiliations:** 1 Department of Radiology, Nagoya City University Graduate School of Medical Sciences, Nagoya, Japan; 2 Department of Integrated Health Sciences, Nagoya University Graduate School of Medicine, Nagoya, Japan; 3 Department of Radiological Technology, Suzuka University of Medical Science, Suzuka, Japan; 4 Department of Gastroenterological Surgery, Nagoya City University Graduate School of Medical Sciences, Nagoya, Japan; Semmelweis University, HUNGARY

## Abstract

**Purpose:**

The purpose of this study was to evaluate the usefulness of turbo spin-echo (TSE) DWI with fusion images in the T-staging compared with T2-weighted imaging (T2WI) alone and conventional echo-planner imaging (EPI) DWI.

**Methods:**

In this prospective study, 4-mm-thick axial EPI-DWI, TSE-DWI, and T2WI were performed with the same slice locations for 20 patients with rectal cancer. Fusion images of DWI and T2WI were created for both EPI-DWI and TSE-DWI. Ten readers independently diagnosed the T-stages and scored the degree of confidence referring to T2WI alone and then to DWI, T2WI, and fusion images (DWI+T2WI) for each EPI-DWI and TSE-DWI. Visual score assessments of image quality were performed for each DWI.

**Results:**

Inter-observer agreement of T-staging for 10 readers was slight on T2WI alone but fair on EPI-DWI+T2WI and excellent on TSE-DWI+T2WI images. No readers gave higher confidence scores for T2WI compared to EPI/TSE-DWI+T2WI and for EPI-DWI+T2WI compared to TSE-DWI+T2WI. In seven pathologically-proven cases, poor, poor to slight, and fair to perfect agreements with the pathological T-stage were observed with T2WI alone, EPI-DWI+T2WI, and TSE-DWI+T2WI, respectively. All readers gave higher scores regarding image distortion and lower scores regarding image noise for TSE-DWI compared to EPI-DWI. For DWI utility, higher scores were assigned for TSE-DWI compared to EPI-DWI in 7 readers and there were no significant differences in the other 3 readers.

**Conclusion:**

TSE-DWI images might be more appropriate for image fusion with T2WI and rectal cancer T-staging compared with EPI-DWI and T2WI alone.

## Introduction

For rectal cancer, preoperative imaging is important for diagnosing the T-stage. In rectal carcinoma patients with regional lymph node metastasis, preoperative neoadjuvant treatment is considered indicated and generally adopted. Whole-body contrast-enhanced CT is the first choice to screen metastasis. Magnetic resonance imaging (MRI) has not been proven to be useful for diagnosis of lymph node metastasis and its scan range is limited. For lymph node-negative patients, the indication depends on the T-stage; preoperative neoadjuvant treatment is accepted for T3 and T4 patients [1–3]. Overstaging of rectal cancer might lead to the overtreatment of T1 and T2 patients, with an increased risk for therapy-related mortality and morbidity [1]. During the past decade, MRI with the phase array surface coil has proven to be the most accurate staging modality. Two-dimensional T2-weighted imaging (T2WI) has been recommended, especially with a high resolution and thinner slices. However, the reported diagnostic accuracy is varied, ranging from 44–100% [2], mainly due to misdiagnoses caused by desmoplastic response around the tumor [1–4]. It has been concluded that gadolinium-enhanced MRI sequences would not improve the diagnostic accuracy for T-staging, because microvessels or reactive tissues could be enhanced similarly to the tumor [1]. Contrast-enhanced CT would also not improve the accuracy for T-staging because of the similar reason and its superiority over MRI has not been proven.

Diffusion-weighted imaging (DWI) explores the random water molecules’ motion, reflecting tumor cellularity. It has been applied to screening and characterization of tumors and monitoring of the response to radiochemotherapy in rectal cancer [5–7]. Some studies assessed the utility of DWI for evaluating the T-stage of rectal cancer [1, 2, 8]. However, Lu, et al. [2] reported that there were no significant differences in diagnostic performance for T-staging between T2WI alone and DWI combined with T2WI. The reason is thought to be that image distortion affected by air might be problematic on conventional DWI using single-shot echo-planar imaging (EPI) [6]. Single-shot turbo spin-echo (TSE) DWI reduces distortion, but the image quality was degraded by blurring and severe image noise. Recently, the parallel imaging technique improved these problems of single-shot TSE-DWI [9, 10]. The utility of TSE-DWI has been reported in evaluations of middle ear, orbital/neck lesions, lung and breast cancers, and hand lesions [11–17]. TSE-DWI may therefore improve T-staging of rectal cancer due to its comparatively low image distortion. However, there have been few TSE-DWI studies applied to T-staging of colon cancer. In addition, to the best of our knowledge, no studies have applied TSE-DWI to image fusion with anatomical MRI (T2WI) and DWI. For conventional EPI-DWI, previous studies have suggested that image fusion of T2WI and DWI can contribute to the detection of primary and recurrent rectal tumors [5, 7]. A previous study on endometrial cancer reported the utility of image fusion for the evaluation of myometrial invasion [18].

The aim of this study was to prospectively evaluate the possibility of TSE-DWI and fused DWI and T2WI images in the local T-staging of primary rectal cancer compared with T2WI alone and conventional EPI-DWI with fusion images.

## Material and methods

### Study design and patients

This study was approved by the Institutional Review Board of Nagoya City University Graduate School of Medical Sciences and Nagoya City University Hospital (No. 60-17-0006). Privacy of the patients was completely protected, and we obtained their oral/written informed consent. This was non-invasive observational study using clinical MR protocol, so oral informed consent was approved by the institutional review board. Eligibility criteria for entry were: (1) pathologically-proven primary rectal cancer; (2) scheduled surgical treatment; (3) no history of pelvic surgery; and (4) agreement to cooperate. The exclusion criteria were: (1) age younger than 18 years; (2) contraindications to MR (incompatible metal implants or pacemakers); (3) final diagnosis of a disease other than rectal cancer, such as a benign lesion, lesion of other origin, and sigmoid cancer. The primary endpoint of the study was assessment of the image quality and diagnostic confidence with point scales. The secondary endpoint was to assess the concordance of T-staging with pathology and among 10 readers. The point scale is rated by an integral number (1, 2, etc.), so a meaningful difference in the score was assumed to be 1. In previous TSE-DWI studies using point scales, the standard deviations (SDs) for the score were up to 0.8 [14, 17]. However, the SD might be higher in pelvic DWI images due to the inhomogeneous magnetic field. To detect a difference of 1 for paired samples, 10 and 14 patients were considered necessary with a power of 0.8 and 2-sided *p* of 0.05 if the SD was assumed to be 1.0 and 1.2, respectively. The SD might exceed 1.0 and we assumed an SD of 1.2. Sample size should be increased by 15% in a non-parametric test, so it was calculated to be 16.1 [19]. Actually, however, we accrued 20 patients for better analysis.

A total of 20 patients (38–79 [median, 68] years old; 14 men and 6 women) entered our study, undergoing MRI between July 2017 and October 2018. Seven patients underwent surgical resection within a month from MRI, and their T-stages were pathologically proven (T1 in 1, T2 in 1, and T3 in 5).

### MRI techniques

All MRI examinations were performed on a 1.5-T or 3-T scanner (Ingenia; Philips Medical Systems, The Netherlands) using a 32-channel dS Torso coil (phase-array body coil). A previous study reported no significant difference between 1.5-T and 3-T MRI for diagnosing the T-stage of rectal cancer [3], so we included examinations with both MRI scanners from the same vendor using similar scan sequences (n = 10 for both scanners). Axial T2WI, EPI-DWI, and TSE-DWI were performed with the same slice location, axial direction, and 4-mm slice thickness, after routinely obtaining images. T2WI and DWI were scanned with the parameters shown in Table 1. T2WI was performed using a two-dimensional TSE sequence. EPI-DWI and TSE-DWI were performed using the single-shot technique and spectral attenuated inversion recovery was used for fat saturation. We did not use antispasmodics, considering the possibility of adverse effects.

**Table 1:** Imaging parameters for T2WI, EPI-DWI, and TSE-DWI.

	1.5-T scanner			3-T scanner		
	T2WI	EPI-DWI	TSE-DWI	T2WI	EPI-DWI	TSE-DWI
Repetition time, ms	5000	6500	6500	5000	7500	7500
Echo time, ms	100	62	61	100	66	63
Flip angle, degrees	90	90	90	90	90	90
Echo train length	17	35	26	21	29	20
b-values, s/mm^2^	-	0, 800	0, 800	-	0, 800	0, 800
Bandwidth, Hz/pixel	189	2984	2830	291	2109	571
Field of view, mm	200	300	300	200	300	300
Matrix size, mm	0.65	3.1	3.1	0.56	3.1	3.1
slice thickness, mm	4	4	4	4	4	4
NSA	2	4	4	1	3	3
SENSE factor	1.9	2.5	2.5	1.4	3	3
Scan time, second	159	189	364	135	172	314

T2WI: T2-weighted imaging, EPI-DWI: echo-planar imaging DWI, TSE-DWI: turbo-spin echo DWI, NSA: number of signals averaged, SENSE: Sensitivity encoding factor.

The fusion images of DWI (high b-value) and T2WI were created with PACS software (EV Insite R, PSP Corporation, Tokyo, Japan) for both EPI-DWI and TSE-DWI. The field of view and slice location of the DWI images were automatically adjusted to exactly match the T2WI, and then the DWI images were converted to a color scale and overlaid on the T2WI images.

### Image data analysis

All evaluations were independently carried out by 10 radiologists (4–13 years of clinical experience), who were blinded to the clinical and pathological information. All image reviews were performed using the PACS software and 3-megapixel monitors (Totoku, Tokyo, Japan) allowing adjustment of the magnification, window, and level settings. Following a previous study [2], images were reviewed in a random order to prevent recall bias. The readers reviewed both DWI and T2WI images with fusion images (DWI+T2WI), and DWI images were visually assessed and scored. For T-staging and diagnostic confidence score, the readers interpreted T2WI alone, EPI-DWI+T2WI, and TSE-DWI+T2WI images in separate viewing sessions at intervals of usually two weeks.

T-staging was based on the 8th Edition criteria of the Japanese Society for Cancer of the Colon and Rectum. Table 2 shows the criteria of pathology and MRI images in this study, referring to previous studies [1, 2]. The degree of diagnostic confidence was scored using a 3-point scale: 1 = unacceptable/possible, 2 = probable, 3 = definite. For visual assessment of EPI-DWI and TSE-DWI images, the degrees of image noise, image distortion, and utility of DWI and fusion images were assessed. The degrees of image noise and distortion were visually scored using a 4-point scale: 1 = unacceptable, 2 = poor, 3 = moderate, 4 = good. The score was assessed as 1 or 2 if artifacts obviously degraded the fusion images. For example, fusion images were judged to be degraded if a colored diffusion-restricted area implying a tumor was presented on normal intensity beyond the low signal intensity area on T2WI due to distortion, or the colored area of the tumor could not be visualized on fusion images due to severe image noise. The degrees of utility of DWI and fusion images were visually scored using a 3-point scale: 1 = not/hardly useful, 2 = useful, 3 = very useful.

**Table 2:** Criteria of pathology and MRI images for T-staging of rectal cancer.

Stage	Criteria of pathology	Criteria of MRI images
T1	Restricted to submucosa	Restricted to submucosal layer, and/or presenting high intensity with a low signal intensity of stalk or thickened component on DWI image
T2	Within muscularis propria	Within muscularis propria
T3	Beyond muscularis propria	Beyond muscularis propria and extending into the perirectal fat
T4a	Penetrating visceral peritoneum	Beyond the mesorectal fascia
T4b	Invading other organs	Invasion to other organs

### Statistical analysis

We used BellCurve for Excel version 2.11 (Social Survey Research Information Co., Ltd., Tokyo, Japan) for statistical analysis. Wilcoxon signed-rank test was used to compare point scale, non-normally distributed ordinal data. Significant difference was set to *p*<0.05. Inter-observer agreement and agreements of T-staging among T2WI alone, DWI+T2WI, and pathology were evaluated using Fleiss’ Kappa statistics. The strength of agreement was evaluated as poor (0.00–0.20), slight (0.21–0.40), fair (0.41–0.60), moderate (0.61–0.80), or excellent or perfect (0.81–1.00).

## Results

**Fig 1:**
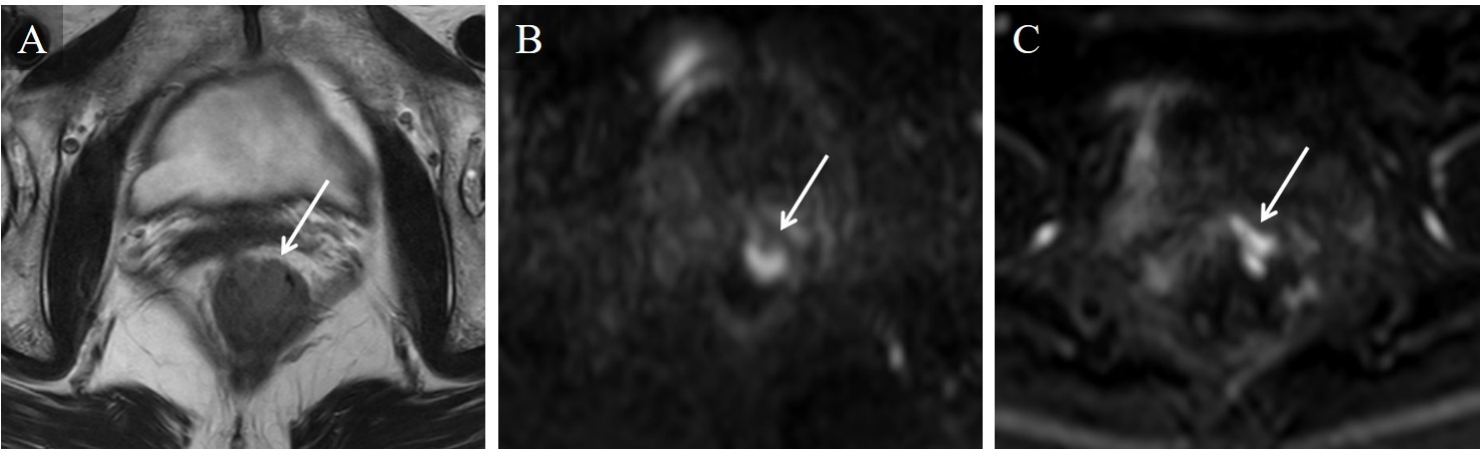
Stage T1 rectal carcinoma in a 75-year-old woman. From the T2WI image (A) alone, all readers misdiagnosed the stage. On the TSE-DWI image (B), an area of high signal intensity with a low signal intensity of stalk was depicted and diagnosed as stage T1. However, this finding was not presented on the EPI-DWI image (C) due to distortion artifacts, and all readers misdiagnosed the stage, as with the T2WI image alone.

**Fig 2:**
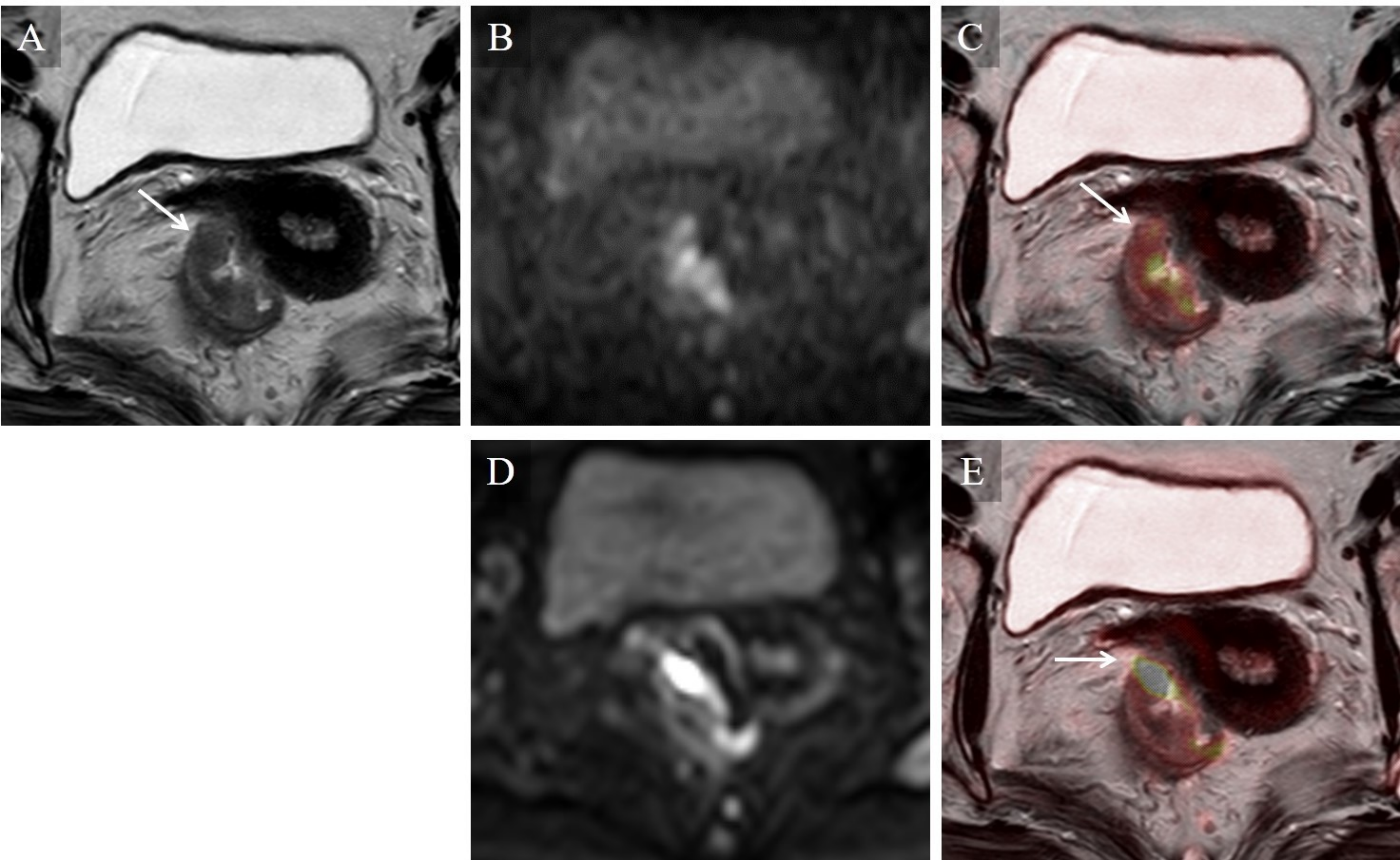
Stage T3 rectal carcinoma in a 69-year-old woman. On the T2WI image (A) alone, intermediate intensity of tumor was shown beyond the muscularis propria (arrow). Higher image noise was seen on the TSE-DWI image (B) than on the EPI-DWI image (D), but this did not degrade the fusion image of TSE-DWI (C). On the fusion image of TSE-DWI (C), a colored area of diffusion restriction corresponded to the low intensity area on the T2WI-presenting tumor (arrow). On the fusion image of EPI-DWI (E), a colored area of diffusion restriction was depicted beyond the low intensity area on T2WI (arrow) due to distortion artifacts, but did not degrade the T-staging.

**Fig 3:**
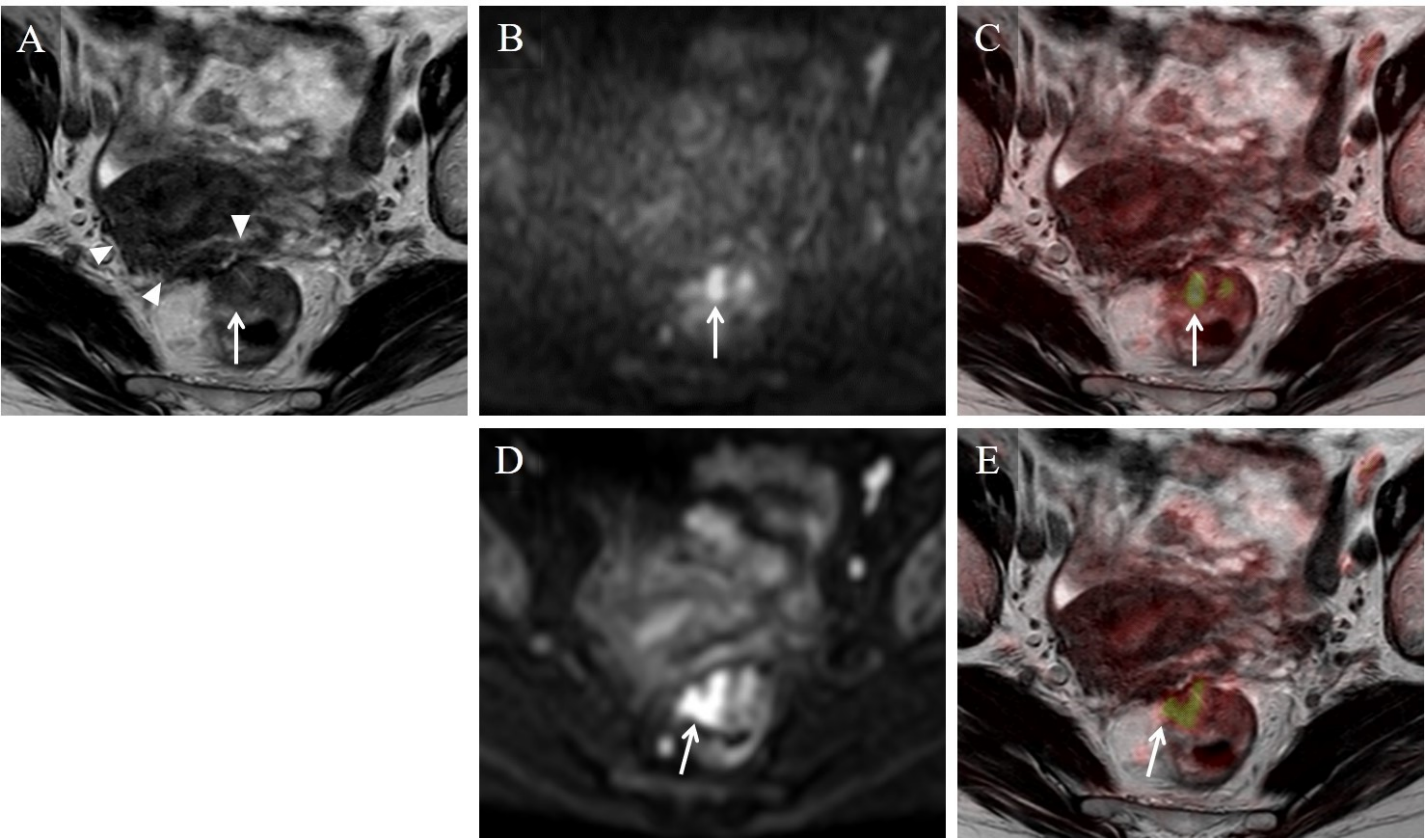
Stage T2 rectal carcinoma in a 75-year-old woman. On the T2WI image (A), a low intensity area extended from the mesorectal fascia into the surface of the uterus (arrow-head). Five readers misdiagnosed the stage as T4a or T4b with T2WI alone. This area did not show high intensity on the DWI image, and was considered as fibrosis. A high intensity lesion was seen on TSE-DWI (B) and EPI-DWI (D) image (arrow). On the fusion image of TSE-DWI (C), 7 readers accurately diagnosed the stage as T2. On the fusion image of EPI-DWI (E), an area of diffusion restriction was shown beyond the muscularis propria (arrow) probably due to image distortion, and the stage was misdiagnosed as T3.

Table 3 shows the inter-observer agreement of T-staging in all cases for T2WI alone, EPI-DWI+T2WI, and TSE-DWI+T2WI and agreement with pathology in 7 cases for each reader. Inter-observer agreement of T-staging among the 10 readers was slight for T2WI alone, fair for EPI-DWI+T2WI, and excellent for TSE-DWI+T2WI (kappa = 0.26, 0.60, and 0.86, respectively). Visual T-staging diagnostic confidence scores are summarized in Table 4, with the numbers of readers assigning a higher score for respective image sequences and those for each median score. No readers gave a higher diagnostic confidence score for T2WI compared to EPI/TSE-DWI+T2WI and for EPI-DWI+T2WI compared to TSE-DWI+T2WI. The median scores of 2, 2, and 3 were assigned in 7, 6, and 8 readers for T2WI, EPI-DWI+T2WI, and TSE-DWI+T2WI, respectively. Poor, poor to slight, and fair to perfect agreements with the pathological T-stage in the 7 cases were observed for T2WI alone, EPI-DWI+T2WI, and TSE-DWI+T2WI, respectively (kappa = 0–0.07, 0–0.22, and 0.60–1, respectively). Representative pathologically-proven cases are presented in Figs 1–3. Table 5 shows the distribution of T-stages diagnosed with the MRI images by the readers for respective pathologically proven T-stages. For pathologically proven T1 stage (Fig 1), 9 readers diagnosed the accurate T-stage on TSE-DWI+T2WI, but all readers misdiagnosed on T2WI alone and EPI-DWI+T2WI. For pathologically proven T3 stage, diagnoses by the readers were more miscellaneous for T2WI alone and EPI-DWI+T2WI compared to TSE-DWI+T2WI, although the accurate diagnosis might not have been difficult in some cases, (e.g., case in Fig 2). For pathologically proven T2 stage (Fig 3), all readers misdiagnosed with T2WI alone and EPI-DWI+T2WI and diagnosed as either T2 or T3 with TSE-DWI+T2WI.

**Table 3:** Inter-observer agreement of T-staging in all 20 cases and agreement between respective reader’s diagnosis and pathology in 7 cases for three image sequences.

Image sequence	Inter-observer agreement for 10 readers (20 cases)	Agreement between pathology and each reader’s diagnosis (7 cases)									
		*1*	*2*	*3*	*4*	*5*	*6*	*7*	*8*	*9*	*10*
T2WI	0.26	0	0	0	0.07	0.02	0	0	0	0	0
EPI	0.60	0.20	0	0.22	0.09	0	0.20	0.22	0	0	0.22
TSE	0.86	1	1	0.67	1	1	0.60	0.60	1	1	0.60

Fleiss’ Kappa values are recorded. Readers are expressed in italic font.

T2WI: T2WI alone, EPI: EPI-DWI and T2WI with fusion images, TSE: TSE-DWI and T2WI with fusion images.

**Table 4:** Comparison of visual T-staging diagnostic confidence scores and distribution of median scores for three image sequences assigned by 10 readers.

**Compared image sequence**	**Higher score on T2WI**		**Higher score on EPI**			**Higher score on TSE**	**No significant differences**
T2WI vs EPI	0		5			-	5
T2WI vs TSE	0		-			8	2
EPI vs TSE	-		0			3	7
	**Median score**				
**Image sequence**	**1**	**2**		**2.5**	**3**
T2WI	1	7		2	0
EPI	0	6		0	4
TSE	0	1		1	8

Values are the number of readers (n = 10).

T2WI: T2WI alone, EPI: EPI-DWI and T2WI with fusion images, TSE: TSE-DWI and T2WI with fusion images.

**Table 5:** T-stages diagnosed by 10 readers in descending order of frequency for 7 pathologically-proven T-stages.

Image sequence	*T1 (n = 1)*	*T2 (n = 1)*	*T3 (n = 5)*
T2WI	T2 T3 T4	T3 T4b T4a	*T3* T2 T4a T4b
EPI	T3 T4b T2 T4a	T3	*T3* T4a
TSE	*T1* T2	*T2* T3	*T3*

Pathologically-proven T-stages are in italic font.

T2WI: T2WI alone, EPI: EPI-DWI and T2WI with fusion images, TSE: TSE-DWI and T2WI with fusion images.

Table 6 summarizes the results of visual score assessment for TSE-DWI and EPI-DWI by the 10 readers and distribution of the median score. For TSE-DWI compared to EPI-DWI, all readers gave higher scores regarding image distortion and lower scores regarding image noise. Regarding DWI utility, 7 readers assigned higher scores for TSE-DWI than EPI-DWI and there were no significant differences for the other 3 readers.

**Table 6:** Assessment of visual image quality of EPI-DWI and TSE-DWI by 10 readers and distribution of median scores.

**Image quality assessment**	**Image sequence showing higher score**			**EPI-DWI**				**TSE-DWI**				
				**Median score**								
	**EPI-DWI**	**TSE-DWI**	**n.s.**	**1**	**2**	**3**	**4**		**1**	**2**	**3**	**4**
Image noise	10	0	0	0	0	0	10		0	0	10	0
Image distortion	0	10	0	0	9	0	1		0	0	0	10
				**Median score**								
				**1**	**1.5**	**2**	**3**		**1**	**1.5**	**2**	**3**
Utility of DWI	0	7	3	2	1	5	2		0	0	1	9

Values are the number of readers (n = 10). n.s.: no significant differences.

## Discussion

Our study demonstrated the utility of TSE-DWI for T-staging of rectal cancer and, to the best of our knowledge, is the first to have applied TSE-DWI to image fusion with T2WI. Diffusion is restricted in the tumor environment, and differentiation between fibrosis and tumor infiltration can be more accurate using DWI [1]. The disadvantages of DWI are low resolution and difficulty in acquiring a positional relationship between the tumor and adjacent normal structures as both show low intensity. To overcome the morphologic ambiguity on DWI images, image fusion of T2WI and DWI has been suggested for the evaluation of various malignant tumors [5, 7, 18]. For the DWI technique using diffusion-sensitive preparation pulse, the single-shot EPI sequence has been used most commonly for more rapid data acquisition to overcome the influence of macroscopic motion while retaining microscopic molecular motion [9, 10]. However, EPI-DWI is sensitive to susceptibility effects, and image distortion causes misregistration on fusion images, especially in adjusting for air or metal. TSE-DWI reduces image distortion, and is thought to potentially be useful in image fusion to evaluate the T-stage of rectal cancer. A 180-degree radiofrequency refocus pulse leads to strong reduction of susceptibility artifacts [9–12, 14, 15]. The main problem with TSE-DWI was slow data acquisition, resulting in motion artifacts and signal decay. Nevertheless, combined use of parallel imaging and single-shot technique, generally used for ultrafast breath-hold T2WI, became applicable to TSE-DWI sequence in recent years, reducing motion artifacts. Single-shot TSE-DWI has issues with severe blurring and image noise, but development of parallel imaging technique and multichannel coil solved these issues [10, 17].

In our study, TSE-DWI showed higher score for image distortion and lower score for image noise in all 10 readers and higher utility score in 7 readers than EPI-DWI. The median score for image noise on TSE-DWI was 3 (fusion images: non-degraded) for all readers and that for image distortion on EPI-DWI was 2 (fusion images: degraded) for 9 readers. So, TSE-DWI might have more advantage for image fusion, although we could not compare these median scores statistically. For the representative cases shown in Figs 1 and 2, higher image noise was seen with TSE-DWI compared to EPI-DWI, but the fusion images were not affected. For the representative case shown in Fig 1, a characteristic finding of T1, stalk appearance [1], was found on TSE-DWI. This finding was thought to represent reactive inflammation and edema lifting the tumor and forming the stalk appearance. However, this finding did not appear on EPI-DWI due to distortion artifacts, leading to misdiagnosis on EPI-DWI+T2WI images. For the representative cases shown in Figs 2 and 3, a colored area of diffusion restriction on the fusion image was affected by distortion artifacts for EPI-DWI. Therefore, TSE-DWI was thought to be useful for fusion with T2WI images compared to conventional EPI-DWI due to the lower image distortion with acceptable image noise.

In the representative case of Fig 3, TSE-DWI improved differentiation between fibrosis and tumor infiltration and prevented overstaging of stage T2. The area of fibrosis widely extended to the surface of the uterus, leading to misdiagnosis as T4 on T2WI images alone. This wide fibrotic area was thought to be caused by another disease such as post-endometriosis. In seven pathologically-proven cases, fair to perfect agreements in T-staging between TSE-DWI+T2WI images and pathology were obtained, and the two cases in Figs 1 and 3 were misdiagnosed on EPI-DWI+T2WI images as discussed in the preceding paragraph. Poor agreement in T-staging between T2WI images alone and pathology was observed. In our study, the remaining 13 cases were not pathologically proven. The reason for this was that preoperative neoadjuvant treatment was performed for T3 and T4 cases [1–3]. Additionally, when the patients were definitely diagnosed as T1 or T4 or as having lymph node metastasis by preoperative CT examination and colon fiberscopy, MRI examination was not performed in our hospital. Nevertheless, for all 20 cases including the pathologically-unproven cases, inter-observer agreement in T-staging for all readers was slight on T2WI alone but excellent on TSE-DWI+T2WI (kappa = 0.26 and 0.86, respectively). In, addition, no readers gave higher confidence scores on T2WI images alone than on EPI/TSE-DWI+T2WI images. Consequently, DWI+T2WI was thought to be at least more useful for T-staging than T2WI alone.

There were several limitations. First, the distribution of tumors at different T-stages was uneven and the sample size was small especially for pathologically-proven cases. The required number of samples was calculated for visual scoring assessment. In addition, this study was not investigational but observational, so the MRI examinations might have been skipped as discussed in the preceding paragraph. Performing a MRI examination without neoadjuvant treatment for all cases may not be ethically justifiable. Second, TSE-DWI requires longer scan time than EPI-DWI. Third, we included examinations from both 1.5-T and 3-T MRI scanners. TSE-DWI might be better suited to a 3-T MRI scanner due to stronger image distortion and less image noise. Fourth, the axial images were obtained in the plane orthogonal to the body axis. T2WI performed in a plane perpendicular to the long axis of the rectum at the level of the tumor was reported to be useful for evaluation of extramural depth tumor invasion [4].

In conclusion, TSE-DWI was more appropriate for image fusion of T2WI and DWI compared to conventional EPI-DWI in terms of less image distortion with acceptable image noise. TSE-DWI+T2WI might be useful compared to T2WI alone and EPI-DWI+T2WI for the T-staging of primary rectal cancer.

## Supporting information

S1 File (PDF)
