# Supplementary material for: T-staging of rectal cancer: Utility of single-shot turbo spin-echo diffusion-weighted imaging with T2-weighted images and fusion images
Source: PLoS One. 2021 Apr 21;16(4):e0249433. doi: 10.1371/journal.pone.0249433 (PMC8059798; doi:10.1371/journal.pone.0249433)
Supplement: S1 File — (PDF) [file pone.0249433.s001.pdf]

Entire visual assessment score

Readers are expressed in italic font.

T staging

	T2										EPI										TSE										Pathology
case No	*1*	*2*	*3*	*4*	*5*	*6*	*7*	*8*	*9*	*10*	*1*	*2*	*3*	*4*	*5*	*6*	*7*	*8*	*9*	*10*	*1*	*2*	*3*	*4*	*5*	*6*	*7*	*8*	*9*	*10*	
1	3	3	3	3	3	3	3	3	3	3	3	3	3	3	3	3	3	3	3	3	3	3	3	3	3	3	3	3	3	3	3
2	2	4a	2	2	3	2	3	2	2	2	3	3	3	3	3	3	3	3	3	3	3	3	3	3	3	3	3	3	3	3	3
3	4a	4b	4a	4a	4a	4b	3	3	3	3	4a	4a	4a	4b	4a	4b	4b	4b	4b	4b	4a	4a	4a	4b	4a	4b	4b	4b	4b	4b	
4	3	4b	4a	2	2	2	2	2	2	3	3	3	2	3	2	2	3	3	3	3	3	3	3	3	3	3	3	3	3	3	
5	2	3	3	2	3	3	3	2	2	3	3	2	3	2	3	3	3	3	3	3	3	3	3	3	3	3	3	3	3	3	
6	3	4a	2	3	2	2	2	2	2	2	3	3	3	3	3	3	3	3	3	3	3	3	3	3	3	3	3	3	3	3	3
7	2	4b	4a	3	3	3	3	2	3	3	3	3	3	3	3	3	3	2	3	3	3	3	3	3	3	3	3	3	3	3	
8	4b	4b	4b	4b	4b	4a	4a	4b	4b	4b	4b	4b	4b	4b	4b	4b	4b	4b	4b	4b	4b	4b	4b	4b	4b	4b	4b	4b	4b	4b	
9	2	3	3	3	3	3	2	3	3	3	3	3	3	3	3	3	3	3	3	3	3	3	3	3	3	3	3	3	3	3	3
10	2	3	3	4a	2	3	2	2	2	2	2	3	4a	4b	3	2	4b	3	3	4b	1	1	2	1	1	1	1	1	1	1	1
11	4a	3	4a	4a	4b	3	3	3	3	4a	3	3	4a	3	3	3	3	3	4a	4a	3	3	3	3	3	3	3	3	3	3	
12	3	4a	3	3	2	3	3	3	3	3	3	3	3	3	3	3	3	3	3	3	3	3	3	3	3	3	3	3	3	3	
13	4a	4a	4b	4b	4b	4b	4b	4b	4b	4b	4b	4b	4b	4b	4b	4b	4b	4b	4b	4b	4b	4b	4b	4b	4b	4b	4a	4b	4b	4b	
14	4a	4b	4a	4a	4a	3	4a	4b	4b	4a	4a	4a	4b	4a	4a	3	4a	4a	4a	4a	4a	4a	4a	4a	4a	4a	4a	4a	4a	4a	
15	4a	4b	4b	4b	4a	3	4a	3	4b	4a	4b	4b	4b	4b	4b	4b	4b	4b	4b	4b	4b	4b	4b	4b	4b	4b	4b	4b	4b	4b	
16	3	3	4a	3	3	3	3	3	4a	3	3	3	3	3	3	3	3	3	3	3	3	3	3	3	3	3	3	3	3	3	
17	3	3	4a	3	2	3	2	3	3	4b	3	3	3	4a	4a	3	3	3	3	3	3	3	3	3	3	3	3	3	3	3	3
18	3	4b	4a	3	4b	3	4a	3	3	4b	3	3	3	3	3	3	3	3	3	3	2	2	2	2	2	3	3	2	2	3	2
19	3	3	3	2	2	2	4a	3	3	3	3	3	3	3	3	2	3	3	3	3	3	3	3	3	3	3	3	3	3	3	
20	2	3	2	2	2	2	2	2	3	3	3	3	3	2	3	2	3	3	3	2	3	3	3	3	3	3	3	3	3	3	

T-staging diagnostic confidence scores

	T2										EPI										TSE									
case No	*1*	*2*	*3*	*4*	*5*	*6*	*7*	*8*	*9*	*10*	*1*	*2*	*3*	*4*	*5*	*6*	*7*	*8*	*9*	*10*	*1*	*2*	*3*	*4*	*5*	*6*	*7*	*8*	*9*	*10*
1	1	1	2	2	2	2	2	2	2	3	3	3	3	2	3	3	2	2	2	3	3	3	3	3	3	2	2	2	3	2
2	2	1	1	1	1	2	2	2	2	1	2	2	2	2	3	2	2	2	2	2	3	3	3	3	3	3	2	2	3	3
3	1	1	2	1	1	3	2	2	3	2	3	3	2	3	2	3	2	3	3	3	3	3	2	3	3	3	2	3	3	3
4	2	2	2	2	2	2	2	2	2	3	2	3	2	2	3	2	2	2	2	2	3	3	3	2	3	2	2	2	3	3
5	1	1	1	2	2	2	2	2	2	3	2	2	2	2	2	2	2	2	3	3	3	3	2	3	3	2	2	2	3	3
6	2	2	1	1	1	3	2	2	2	2	3	3	2	2	2	2	2	2	3	3	3	3	2	3	2	2	2	2	3	3
7	2	1	2	2	2	3	2	3	2	3	3	3	2	3	3	3	2	2	2	3	3	3	3	2	3	3	2	3	2	3
8	3	3	3	3	3	2	2	3	3	3	3	3	3	3	3	3	2	3	3	3	3	3	3	3	3	3	2	3	3	3
9	2	3	2	2	2	2	2	2	3	3	3	3	3	3	2	3	2	2	3	3	3	3	3	2	3	3	2	2	3	3
10	1	1	2	1	2	2	2	2	2	2	2	3	2	2	1	2	2	3	2	1	2	2	3	2	3	3	2	3	3	2
11	1	1	2	1	2	2	2	2	3	1	3	3	2	2	2	3	2	3	2	3	3	3	3	2	3	3	2	2	2	2
12	1	1	2	2	1	2	2	2	3	3	3	3	3	2	3	3	2	3	2	3	3	3	3	2	3	2	2	2	3	3
13	2	1	3	3	2	3	2	3	3	3	2	2	3	3	3	3	3	3	3	3	2	3	3	3	3	3	2	3	3	3
14	2	3	2	3	2	3	2	2	2	2	2	2	2	2	2	3	2	2	2	3	3	2	2	3	3	3	2	3	3	3
15	2	2	1	2	1	3	2	2	3	3	3	3	2	2	3	3	2	3	3	3	2	2	2	2	3	3	2	3	3	3
16	2	2	1	2	2	3	2	2	2	2	3	3	3	3	2	3	2	2	3	3	3	3	3	2	3	3	2	3	3	3
17	1	2	1	1	2	2	2	2	3	2	3	3	2	2	1	3	2	3	2	2	3	3	3	3	2	3	2	3	3	3
18	1	1	2	1	2	2	2	2	3	2	3	3	2	2	2	2	2	2	2	2	2	2	2	2	3	3	2	3	2	3
19	2	2	2	1	2	2	2	3	3	3	3	3	2	1	2	2	2	3	3	3	3	3	3	3	2	3	2	3	3	3
20	1	1	1	1	1	3	2	2	2	2	3	3	2	1	2	2	2	2	2	2	3	3	2	2	2	3	2	2	2	3

T2WI: T2WI alone, EPI: EPI-DWI and T2WI with fusion images, TSE: TSE-DWI and T2WI with fusion images.

Image quality scores of DWI

case No 1

	Image noize		Image distortion		Utility of DWI	
	TSE-	EPI-	TSE-	EPI-	TSE-	EPI-
1	3	4	4	2	3	2
2	3	4	4	2	3	1
3	2	4	4	2	2	1
4	4	4	4	2	3	1
5	2	4	4	1	2	1
6	2	4	4	3	2	2
7	3	4	4	2	3	2
8	3	4	4	2	3	2
9	3	4	4	2	3	1
10	3	4	4	2	3	1
11	3	4	4	3	3	3
12	2	3	4	2	2	1
13	4	4	4	3	3	2
14	3	4	4	3	3	2
15	2	3	4	2	2	2
16	3	4	4	2	3	2
17	3	4	4	2	3	1
18	3	4	4	2	3	1
19	3	4	4	2	3	1
20	3	4	4	2	3	1

case No 2

	Image noize		Image distortion		Utility of DWI	
	TSE-	EPI-	TSE-	EPI-	TSE-	EPI-
1	3	4	4	2	3	2
2	3	4	4	2	3	2
3	2	3	4	2	2	1
4	4	4	4	2	3	1
5	2	4	4	1	2	1
6	2	4	4	2	2	2
7	3	4	4	2	3	2
8	3	4	4	2	3	1
9	3	4	4	2	3	1
10	3	4	4	2	3	1
11	4	4	4	4	3	3
12	2	4	4	2	3	1
13	4	4	4	3	3	2
14	3	4	4	3	3	2
15	3	3	4	2	3	2
16	3	4	4	2	3	2
17	3	4	4	2	3	1
18	3	4	4	2	3	1
19	3	4	4	2	3	1
20	3	3	4	2	3	1

case No 3

	Image noize		Image distortion		Utility of DWI	
	TSE-	EPI-	TSE-	EPI-	TSE-	EPI-
1	3	4	4	2	2	2
2	3	4	4	2	3	2
3	3	4	4	2	2	2
4	3	4	4	2	3	2
5	3	4	3	1	2	1
6	2	4	4	3	2	2
7	3	4	4	3	3	3
8	3	4	4	2	3	2
9	3	4	4	2	3	2
10	3	4	4	2	3	1
11	3	4	4	3	3	3
12	3	4	4	2	3	2
13	3	4	4	3	2	2
14	3	4	4	3	2	2
15	3	4	4	2	2	2
16	3	4	4	3	3	3
17	3	4	4	2	3	1
18	3	4	3	2	3	1
19	3	4	4	2	2	2
20	3	4	4	2	2	2

case No 4

	Image noize		Image distortion		Utility of DWI	
	TSE-	EPI-	TSE-	EPI-	TSE-	EPI-
1	3	4	4	2	2	2
2	3	4	4	3	3	2
3	3	2	4	3	3	1
4	3	3	4	2	3	1
5	3	4	4	1	3	1
6	3	4	4	4	2	2
7	3	4	4	2	3	2
8	3	4	4	3	2	2
9	3	4	4	2	2	2
10	3	3	4	2	3	1
11	3	4	4	2	3	1
12	2	3	4	2	2	2
13	4	4	4	3	2	2
14	3	4	4	3	3	2
15	2	3	4	3	2	2
16	3	3	4	3	3	2
17	3	3	4	2	3	1
18	2	4	4	2	3	1
19	3	4	4	2	3	1
20	2	3	4	3	2	2

case No 5

	Image noize		Image distortion		Utility of DWI	
	TSE-	EPI-	TSE-	EPI-	TSE-	EPI-
1	2	4	4	2	2	2
2	3	4	4	3	3	2
3	3	3	4	2	2	3
4	3	4	4	2	2	2
5	3	3	4	1	2	1
6	3	3	4	3	2	3
7	3	4	4	2	3	2
8	3	4	4	3	3	3
9	3	4	4	2	3	2
10	3	1	4	1	3	1
11	3	4	3	3	3	2
12	3	4	4	2	3	2
13	3	4	4	4	3	3
14	4	4	4	4	3	3
15	2	4	4	4	2	3
16	4	4	4	2	3	2
17	3	4	4	2	3	2
18	3	4	4	2	3	2
19	3	4	4	2	3	2
20	2	4	4	2	2	2

case No 6

	Image noize		Image distortion		Utility of DWI	
	TSE-	EPI-	TSE-	EPI-	TSE-	EPI-
1	3	4	4	2	2	2
2	3	4	4	4	2	2
3	3	4	4	2	2	2
4	3	4	4	2	3	2
5	3	3	4	1	2	1
6	3	4	4	4	2	2
7	3	4	4	4	3	2
8	3	4	4	4	3	2
9	3	4	4	2	2	2
10	3	4	3	2	3	2
11	3	4	4	3	3	2
12	3	4	4	4	2	2
13	3	4	4	4	2	2
14	3	4	4	4	3	2
15	3	4	4	4	2	2
16	3	4	4	4	2	2
17	3	4	4	3	2	2
18	3	4	4	4	2	2
19	3	4	4	4	2	2
20	3	4	4	4	2	3

case No 7

	Image noize		Image distortion		Utility of DWI	
	TSE-	EPI-	TSE-	EPI-	TSE-	EPI-
1	3	4	4	2	3	2
2	3	4	4	2	3	3
3	3	4	4	2	2	2
4	3	4	4	2	3	3
5	3	4	3	1	2	1
6	3	4	4	4	2	3
7	3	4	4	2	3	3
8	3	4	4	4	3	3
9	3	4	4	2	3	3
10	3	4	4	2	3	1
11	3	4	4	2	3	2
12	2	4	4	2	2	2
13	4	4	4	2	3	2
14	3	4	4	2	3	3
15	3	4	4	2	2	3
16	3	4	4	2	3	3
17	3	4	4	2	3	2
18	3	4	4	2	3	2
19	3	4	4	2	3	3
20	3	4	4	2	2	3

case No 8

	Image noize		Image distortion		Utility of DWI	
	TSE-	EPI-	TSE-	EPI-	TSE-	EPI-
1	3	4	4	2	3	3
2	3	4	4	2	3	2
3	3	4	4	2	3	3
4	4	4	4	2	3	2
5	3	4	4	1	3	1
6	3	4	4	3	2	2
7	3	4	4	2	3	2
8	3	4	4	2	3	2
9	3	4	4	2	3	2
10	3	4	4	2	3	1
11	3	4	4	3	3	3
12	2	4	4	2	2	2
13	4	4	4	4	3	3
14	3	4	4	4	3	3
15	3	4	4	3	2	3
16	3	4	4	3	3	3
17	3	4	4	2	3	2
18	3	4	4	2	3	2
19	3	4	4	2	3	2
20	3	4	4	2	2	2

case No 9

	Image noize		Image distortion		Utility of DWI	
	TSE-	EPI-	TSE-	EPI-	TSE-	EPI-
1	3	4	2	2	3	3
2	3	4	4	2	3	3
3	3	4	3	2	3	3
4	3	4	4	3	3	3
5	2	4	3	1	2	1
6	3	4	4	3	2	2
7	3	4	4	2	3	2
8	3	4	4	3	3	3
9	3	4	4	3	3	3
10	3	4	4	2	3	1
11	3	4	4	3	3	3
12	3	4	4	2	3	3
13	4	4	4	4	3	3
14	4	4	4	4	3	3
15	3	4	4	4	3	3
16	3	4	4	3	3	3
17	3	4	4	2	3	2
18	3	4	4	2	3	2
19	3	4	4	2	3	3
20	3	4	4	2	2	3

case No 10

	Image noize		Image distortion		Utility of DWI	
	TSE-	EPI-	TSE-	EPI-	TSE-	EPI-
1	3	4	4	3	2	2
2	3	4	4	2	3	2
3	3	4	4	2	3	1
4	4	4	4	3	3	2
5	3	4	4	1	2	1
6	3	4	4	3	2	2
7	3	4	4	2	3	2
8	3	4	4	2	3	2
9	3	4	4	2	3	1
10	3	4	4	2	3	1
11	3	4	4	2	3	1
12	3	4	4	2	2	1
13	4	4	4	4	3	3
14	3	4	4	2	3	3
15	3	4	4	4	2	3
16	3	4	4	2	3	1
17	3	4	4	2	3	1
18	3	4	4	2	3	1
19	3	4	4	2	3	2
20	3	4	4	2	2	1

TSE-: TSE-DWI, EPI-: EPI-DWI.
